# Effectiveness of an Essential Oil Mouthwash on Halitosis in Obese Patients with Periodontitis: A Short-Term Clinical Evaluation

**DOI:** 10.3390/jcm14155225

**Published:** 2025-07-23

**Authors:** Gabriela Beresescu, Despina Luciana Bereczki-Temistocle, Liana Beresescu, Alina Ormenisan, Adriana Monea, Ion Razvan-Marius

**Affiliations:** 1 Faculty of Dental Medicine, George Emil Palade University of Medicine, Pharmacy, Science, and Technology of Targu Mures, 540139 Targu Mures, Romaniaalina.ormenisan@umfst.ro (A.O.); 2 Doctoral School of Medicine and Pharmacy, George Emil Palade University of Medicine, Pharmacy, Sciences and Technology of Targu Mures, 540139 Targu Mures, Romania; 3 2nd Department of Surgery, Mures County Emergency Hospital, 540136 Targu Mures, Romania

**Keywords:** halitosis, periodontitis, obesity, essential oil mouthwash, oral hygiene, BANA test, organoleptic score

## Abstract

**Background:** Halitosis is a common condition often rooted in periodontal disease and exacerbated by systemic disorders such as obesity. This short-term clinical evaluation investigates the relationship between halitosis, obesity, and periodontitis, and assesses the efficacy of a natural essential oil mouthwash as an adjunctive oral hygiene intervention. **Methods:** In this randomized, placebo-controlled clinical trial, 45 obese patients with diagnosed periodontitis and self-reported halitosis were randomly assigned to either a test group (n = 30), receiving an essential oil-based mouthwash, or a control group (n = 15), receiving a placebo. Over 28 days, participants were evaluated using plaque index (PI), bleeding on probing (BOP), organoleptic scoring, and BANA test results. Both subjective and objective halitosis assessments were performed. **Results:** The test group showed marked improvements in all parameters compared to controls. PI decreased by 31.5% in the test group versus 9.25% in controls; BOP reduced by 34.5% versus 6.0%; BANA test positivity dropped by 38.1% in the test group. Organoleptic scores improved by 45.9% (examiner-rated) and 36.8% (self-assessed) in the test group. **Conclusions:** This 28-day clinical evaluation demonstrates the potential of an essential oil-based mouthwash to significantly reduce halitosis and periodontal inflammation in obese individuals with periodontitis. The necessity of future randomized trials is evident to substantiate the sustained benefits and safety of the intervention.

## 1. Introduction

Halitosis, or oral malodor, is a common condition that adversely affects social interactions, quality of life, and psychological well-being. It is reported to affect 30–50% of the global population, with a higher prevalence noted among individuals with systemic comorbidities such as obesity and diabetes [1,2]. While frequently perceived as a cosmetic issue, halitosis often signifies underlying oral or systemic pathology, particularly in patients with poor oral hygiene or chronic inflammatory conditions [3].

Intraoral halitosis, the most common type, is predominantly caused by volatile sulfur compounds (VSCs) such as hydrogen sulfide, methyl mercaptan, and dimethyl sulfide. These malodorous compounds arise from the proteolytic degradation of sulfur-containing amino acids by anaerobic Gram-negative bacteria located on the tongue dorsum and within periodontal pockets [4,5,6]. The key microbial species implicated in VSC production include *Porphyromonas gingivalis*, *Treponema denticola*, and *Tannerella forsythia*—collectively known as the “red complex” and major contributors to both halitosis and periodontal disease [7,8].

Periodontitis, a chronic inflammatory disease that affects the tissues that support teeth, is among the primary intraoral causes of halitosis. The condition is marked by gingival inflammation, connective tissue destruction, alveolar bone loss, and the formation of periodontal pockets. These pockets serve as optimal anaerobic niches for the proliferation of virulence factors produced by VSC-producing pathogens [9,10]. The most recent classification system proposed by the European Federation of Periodontology (EFP) and the American Academy of Periodontology (AAP) delineates four stages and three grades of periodontitis, with the staging system incorporating clinical attachment loss, disease progression, and modifying risk factors such as smoking and obesity [11].

It is now widely recognized that obesity is not only a systemic condition but also functions as a significant modifier of risk for periodontal disease and oral dysbiosis. It is well established that the pathophysiological mechanisms that link obesity and periodontitis are driven by the presence of low-grade, chronic systemic inflammation, which is initiated by proinflammatory cytokines derived from adipose tissue. These include, but are not limited to, tumor necrosis factor alpha (TNF-α), interleukin 6 (IL-6), and leptin [12,13,14]. These mediators have been demonstrated to contribute to altered immune responses, delayed tissue repair, and increased susceptibility to infection, all of which have been shown to worsen periodontal outcomes. Furthermore, obesity-related comorbidities such as type 2 diabetes and gastroesophageal reflux disease (GERD) can further exacerbate halitosis via xerostomia, increased salivary glucose, and acid reflux into the oral cavity [15,16,17].

Emerging research has begun to explore the gut–oral axis and the potential role of probiotics and prebiotics in modulating oral microbiota and managing halitosis [18,19]. The oral administration of beneficial bacterial strains—such as *Lactobacillus salivarius* or *Streptococcus salivarius*—has shown promise in reducing VSC levels and improving breath odor in small-scale trials. These findings underscore the relevance of microbial modulation in halitosis management, especially in patients with systemic dysbiosis, such as those with obesity [13,14].

From a therapeutic standpoint, chlorhexidine (CHX) remains a gold standard for chemical plaque control and halitosis reduction. However, its long-term use is often limited by adverse effects such as mucosal irritation, tooth staining, and altered taste perception [20]. As a result, there is growing interest in natural alternatives, including essential oil-based mouthwashes, which offer antimicrobial, anti-inflammatory, and antioxidant properties with minimal side effects [21,22]. Essential oils such as lemon (*Citrus limon*), melissa (*Melissa officinalis*), and grapefruit seed extract have demonstrated inhibitory effects on VSC-producing bacteria and periodontal pathogens in vitro and in clinical settings [23,24,25]. Their incorporation into oral hygiene regimens may be particularly beneficial in patients seeking non-pharmacological options or those at an elevated risk of systemic inflammation.

Despite promising preliminary data, most available studies have focused on healthy individuals or small populations. There remains a paucity of well-designed, placebo-controlled clinical trials evaluating the efficacy of essential oil mouthwashes in high-risk populations—particularly obese patients with periodontitis. Additionally, little is known about the short-term and long-term effectiveness of such interventions on clinical indices of periodontal inflammation and halitosis under real-world conditions.

The objective of the present study is to evaluate the short-term clinical effectiveness of an experimental essential oil mouthwash in reducing halitosis and improving periodontal health parameters in obese patients with chronic periodontitis. The study employs both subjective and objective halitosis assessment methods, microbiological testing (BANA assay), and validated clinical indices (plaque index, bleeding on probing) over a 28-day intervention period.

## 2. Materials and Methods

This study was designed as a short-term, prospective, randomized, clinical trial aimed at evaluating the effectiveness of a natural essential oil-based mouthwash in reducing halitosis among obese patients diagnosed with chronic periodontitis (according with EFP 2017 guidelines). The study duration was 28 days and focused on assessing both clinical and microbiological parameters. The protocol was approved by the Ethics Committee of the George Emil Palade University of Medicine, Pharmacy, Science, and Technology of Targu Mures, Romania (Approval No. 1863/15.09.2022). All procedures were conducted in accordance with the Declaration of Helsinki, and written informed consent was obtained from all participants.

### 2.1. Participants and Eligibility Criteria

Patients were recruited from the university dental clinic between October 2022 and February 2023. Eligible participants were aged between 25 and 65 years and met the following inclusion criteria:

- Obesity, defined as a body mass index (BMI) ≥ 30 kg/m^2^, classified according to World Health Organization (WHO) thresholds (Class I: 30–34.9 kg/m^2^; Class II: 35–39.9 kg/m^2^; Class III: ≥40 kg/m^2^).
- Diagnosis of chronic periodontitis, confirmed by a periodontist and classified according to the EFP 2017 guidelines.
- Poor oral hygiene, indicated by a plaque index ≥10% and gingival index ≥ 10%.
- Self-reported halitosis.

Exclusion criteria included the presence of xerostomia, active carious lesions or apical pathology, antibiotic or antiseptic mouthwash use in the previous six months, smoking within 48 h prior to assessment, uncontrolled systemic diseases, or pregnancy.

**Figure 1:**
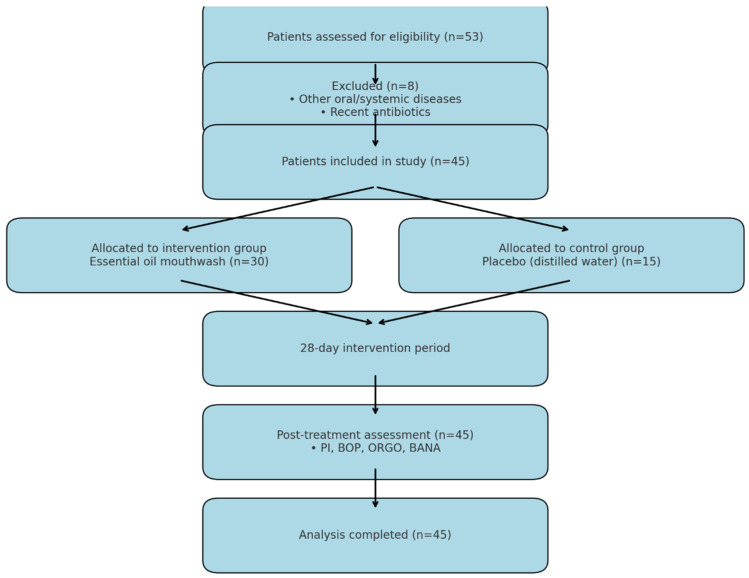
The flow chart of the study.

Out of 53 initially screened individuals, 45 met the inclusion criteria and were randomized into two groups: 30 participants in the test group received the essential oil-based mouthwash, while 15 participants in the control group received a placebo rinse (distilled water). Participants were randomly allocated at a 2:1 ratio into test (n = 30) or control (n = 15) groups, justified by feasibility constraints and a focus on capturing variability in the experimental treatment (Figure 1). Randomization was performed using a computer-generated sequence. The unequal group sizes reflected feasibility and a focus on detecting variability in the experimental intervention.

### 2.2. Intervention Protocol

All participants received standardized oral hygiene instructions and were provided with identical toothbrushes and fluoridated toothpaste.

The test group was assigned a natural experimental mouthwash containing a blend of essential oils, while the control group received distilled water. Participants were instructed to rinse with 10 mL of their assigned solution twice daily for 30 s after brushing.

The experimental essential oil mouthwash was prepared in a sanitized laboratory setting and consisted of the following ingredients: 250 mL of distilled water, 0.5 g of sodium bicarbonate, 2 drops of *Citrus limon* (lemon) essential oil, 2 drops of *Melissa officinalis* (melissa) essential oil, 5 mL of vegetable glycerine as an emulsifier, and 10 drops of grapefruit seed extract as a natural preservative. All components were gently mixed and stored in sterile amber glass bottles to prevent photodegradation. No adverse reactions were reported during the intervention period. The placebo consisted of distilled water matched closely in sensory properties (appearance, taste, and smell), packaging, and labelling to ensure blinding effectiveness. Both mouthwashes were freshly prepared weekly and refrigerated at 4 °C to maintain their stability during the study period.

### 2.3. Clinical Assessment

Clinical assessments were performed at baseline and after 28 days by a single calibrated periodontist (κ = 0.86), ensuring consistency in data collection. Examiner calibration included repeated intra-examiner agreement tests prior to study initiation. The following indices were recorded:

- Plaque Index (PI): Assessed using the O’Leary plaque control record, examining representative vestibular and lingual surfaces of molars and incisors.
- Gingival Index (GI): Evaluated using the Löe and Silness index.
- Bleeding on Probing (BOP): Recorded as the percentage of sites exhibiting bleeding upon gentle probing.

### 2.4. Halitosis Evaluation

Halitosis was assessed using both objective and subjective methods:

Organoleptic Measurement: Examiner-rated odor was scored according to Rosenberg’s 0–5 scale. Participants were asked to refrain from eating strong-smelling foods, using mouthwash, or wearing scented products 48 h prior to the examination. The procedure involved the participant keeping their mouth closed for three minutes and then exhaling slowly toward the examiner from a 10 cm distance. The examiner, blinded to group allocation, rated the odor.

Self-Perception Test: Participants were instructed to perform the wrist-lick test and rate their perceived odor on the same 0–5 scale.

### 2.5. Microbiological Analysis

To assess bacterial load, samples were taken from the dorsal surface of the tongue using sterile swabs. The presence of volatile sulfur compound (VSC)-producing anaerobes—*Porphyromonas gingivalis*, *Treponema denticola*, and *Tannerella forsythia*—was detected using the BANA (benzoyl-DL-arginine-naphthylamide) enzymatic assay. Test strips were moistened, incubated at 55 °C for five minutes, and evaluated for color change, which was interpreted as follows:

- Negative: No color change.
- Slight Positive: Light blue speckling.
- Positive: Uniform strong blue color.

This method, although semi-quantitative, offers a practical and validated tool for clinical detection of VSC-producing pathogens associated with halitosis and periodontal disease.

### 2.6. Statistical Analysis

Data analysis was performed using SPSS version 25.0 (IBM Corp., Armonk, NY, USA). The Shapiro–Wilk test was applied to evaluate the normality of distribution. Parametric tests (paired and independent *t*-tests) were used for normally distributed data, while non-parametric tests (Wilcoxon signed-rank) were used for skewed variables. A *p*-value < 0.05 was considered statistically significant.

## 3. Results

The findings of the present study demonstrate the efficacy of an essential oil mouthwash in mitigating halitosis, as well as its impact on the plaque index, the bleeding index on probing, the bacterial concentration, and the organoleptic examination conducted by both the examiner and the subject. The preliminary findings suggested significant alterations after treatment, with a conspicuous incongruity detected in the test group regarding the plaque index, bleeding on probing, gingival index, BANA test outcomes, and organoleptic examination values.

Initially, a total of 53 patients were assessed for eligibility. Following the screening process, 45 obese individuals afflicted with chronic periodontitis and self-reported halitosis were included in the study and randomly assigned to either the test group (n = 30) or the control group (n = 15) at a 2:1 ratio. All participants completed the 28-day study protocol.

A subsequent demographic analysis confirmed that the baseline characteristics of the two groups were comparable. The mean age was 43.2 ± 9.4 years in the test group and 42.7 ± 8.8 years in the control group (*p* = 0.81). The BMI values were found to be comparable as well (32.1 ± 2.4 vs. 31.7 ± 2.3; *p* = 0.63). The prevalence of smoking was comparable in both groups, with a proportion of 26.7% across both groups. No statistically significant disparities were identified in terms of gender distribution, medical history, or initial periodontal indices.

Demographic analysis confirmed comparable baseline characteristics between groups. The mean age was 43.2 ± 9.4 years in the test group and 42.7 ± 8.8 in the control group (*p* = 0.81). BMI values were also similar (32.1 ± 2.4 vs. 31.7 ± 2.3; *p* = 0.63). Smoking prevalence was equivalent in both groups (26.7%), and no significant differences were observed in gender distribution, medical history, or baseline periodontal indices (Table 1).

**Figure 2:**
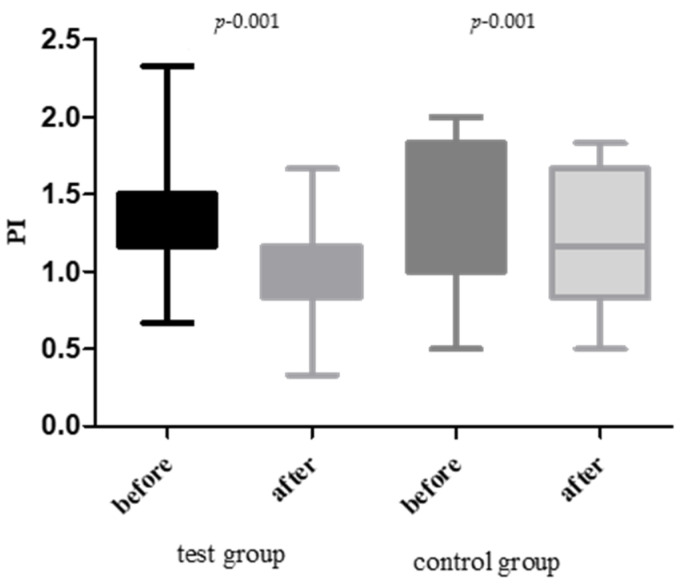
Plaque index before and after treatment in both groups.

A comparison of the test and control groups revealed a statistically significant difference in the plaque index (PI), with the test group demonstrating a more pronounced change. There was an average plaque index of 1.333 in the control group before treatment. At baseline, the mean PI was 1.39 ± 0.15 in the test group and 1.33 ± 0.13 in the control group (*p* = 0.17). After 28 days, the test group showed a statistically significant reduction in PI to 0.95 ± 0.14, representing a 31.5% decrease (*p* < 0.001). The control group exhibited a modest reduction from 1.33 to 1.21 (↓ 9.25%; *p* = 0.041). A between-group comparison confirmed a significantly greater PI reduction in the test group (*p* < 0.001) (Figure 2).

**Figure 3:**
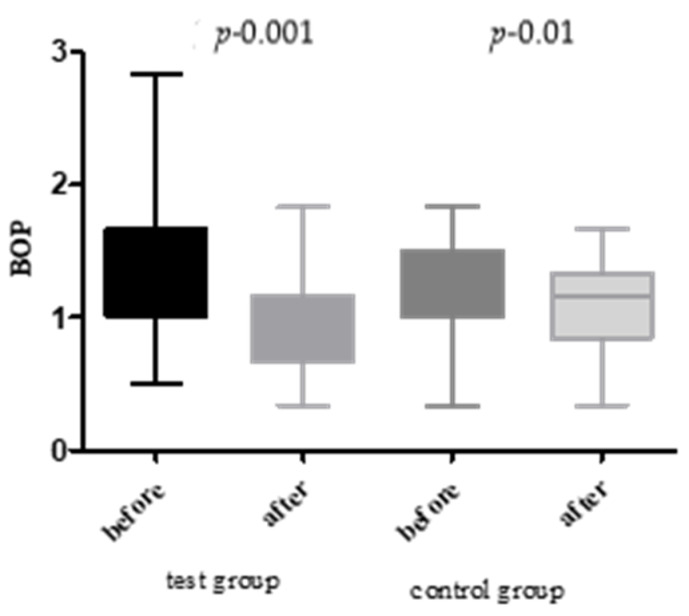
BOP before and after treatment in both groups.

The baseline bleeding on probing (BOP) value was 58.2 ± 6.1% in the test group and 56.4 ± 5.7% in the control group (*p* = 0.31). After the intervention, the test group BOP decreased significantly to 38.1 ± 5.2% (↓ 34.49%; *p* < 0.001). In contrast, the control group exhibited a smaller, non-significant change to 53.0 ± 5.5% (↓ 6.02%; *p* = 0.18). Intergroup analysis showed a statistically significant improvement in BOP in the test group compared to the control group (*p* < 0.001) (Figure 3).

**Figure 4:**
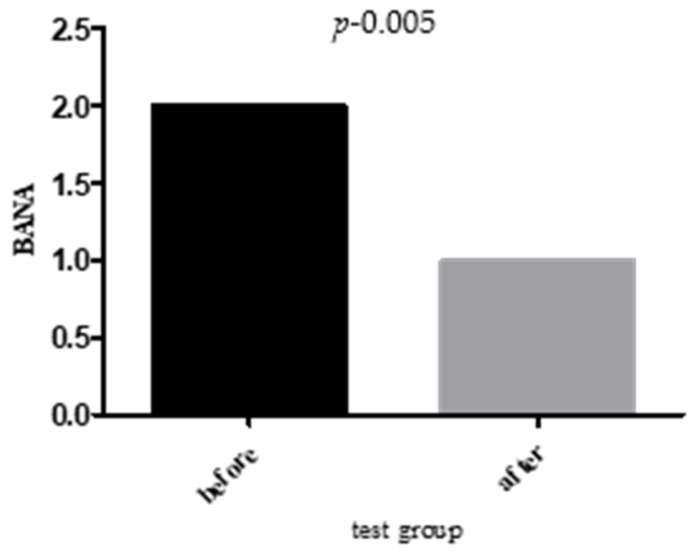
Changes in BANA test before and after treatment in test group.

The BANA test, which detects the enzymatic activity of *P. gingivalis*, *T. forsythia*, and *T. denticola*, revealed substantial microbiological changes. At baseline, 77% of the test group had positive BANA results, which decreased to 38% after 28 days (↓ 38.09%; *p* < 0.001). In the control group, BANA positivity dropped slightly from 68% to 65% (↓ 4.38%; *p* = 0.67). The between-group comparison was statistically significant (*p* < 0.001), indicating superior bacterial suppression in the test group (Figure 4).

**Figure 5:**
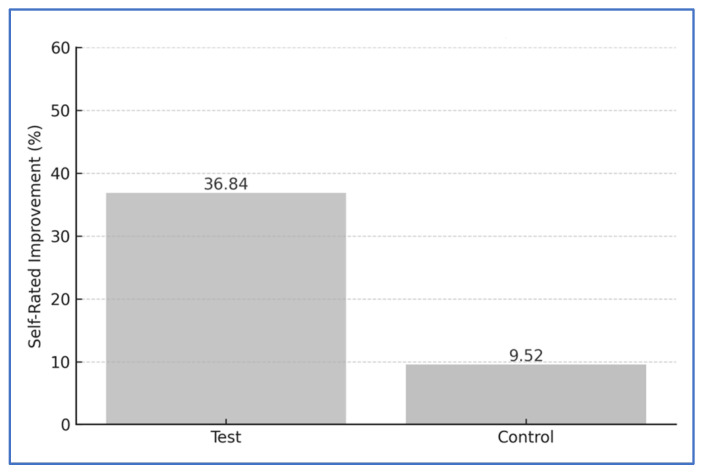
Self-rated halitosis improvement.

Subjective (self-assessed) halitosis scores were evaluated using the wrist-lick test, also on a 0–5 scale. The test group reported a significant decrease from 3.4 ± 0.7 to 2.1 ± 0.6 (↓ 36.84%; *p* < 0.001). The control group showed a smaller, non-significant reduction from 3.2 ± 0.6 to 2.9 ± 0.5 (↓ 9.52%; *p* = 0.13). The difference between groups was statistically significant (*p* < 0.001) (Figure 5).

**Figure 6:**
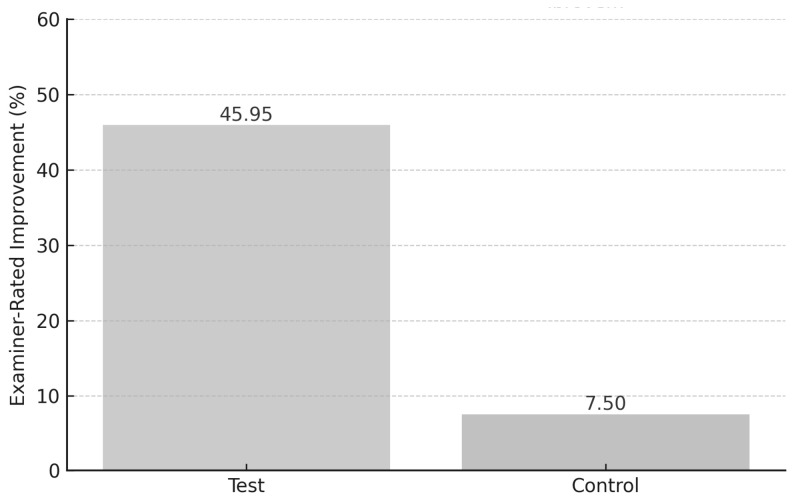
Examiner-rated halitosis Improvement.

Objective (organoleptic) scores were evaluated using a standardized examiner-rated scale from 0 (no odor) to 5 (severe odor). The mean baseline score in the test group was 3.1 ± 0.6, which significantly decreased to 1.7 ± 0.5 after the intervention (↓ 45.95%; *p* < 0.001). The control group showed only a minor change from 3.0 ± 0.5 to 2.8 ± 0.4 (↓ 7.5%; *p* = 0.21). The intergroup difference was significant (*p* < 0.001) (Figure 6).

**Figure 7:**
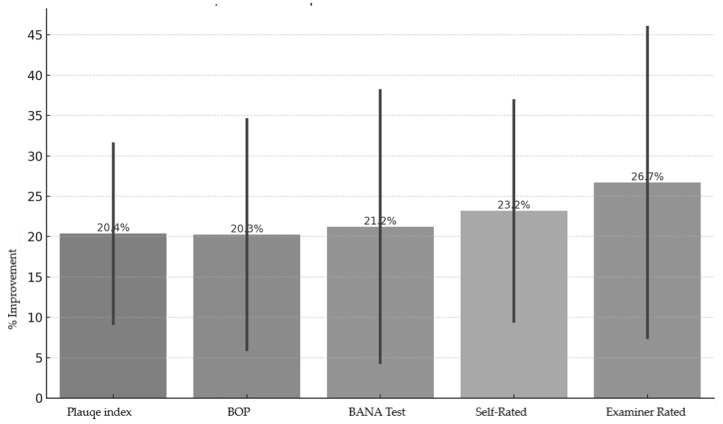
Comparative improvement in clinical parameters after 28 days.

Figure 7 illustrates the percentage change across five core parameters (PI, BOP, BANA, self-reported halitosis, and examiner-rated halitosis). In each category, the test group demonstrated superior improvements compared to the control group:

- Plaque Index: ↓ 31.5% (test) vs. ↓ 9.25% (control).
- BOP: ↓ 34.49% (test) vs. ↓ 6.02% (control).
- BANA Positivity: ↓ 38.09% (test) vs. ↓ 4.38% (control).
- Self-reported halitosis: ↓ 36.84% (test) vs. ↓ 9.52% (control).
- Examiner-rated halitosis: ↓ 45.95% (test) vs. ↓ 7.5% (control).

All participants reported adherence to the rinse protocol. No participants experienced mucosal irritation, altered taste, or other adverse effects. Mouthwash compliance was confirmed through returned volume and self-reporting forms.

The results clearly indicate that the essential oil mouthwash was significantly more effective than the placebo in reducing plaque, gingival inflammation, halitosis (both subjective and objective), and microbial burden. These findings support the inclusion of essential oil rinses as an adjunctive therapy in the oral hygiene regimen of obese patients with periodontitis and halitosis.

## 4. Discussion

The present short-term clinical study demonstrates that a natural essential oil mouthwash significantly reduces halitosis and improves periodontal health among obese patients with periodontitis over a 28-day period. The test group exhibited significant improvements across all measured domains—including plaque index (PI), bleeding on probing (BOP), microbiological profiles (BANA test), and halitosis scores—when compared to the placebo group. These findings support the short-term clinical effectiveness and tolerability of essential oils as adjuncts in the management of oral halitosis; halitosis is a multifactorial condition with predominant intraoral aetiology, especially among patients with periodontal disease. Anaerobic bacteria such as *Porphyromonas gingivalis*, *Treponema denticola*, and *Tannerella forsythia* metabolize sulfur-containing amino acids to produce volatile sulfur compounds (VSCs), including hydrogen sulfide and methyl mercaptan. These compounds chiefly contribute to oral malodor [1,6,26,27]. These microorganisms thrive in periodontal pockets and tongue coatings, where oxygen tension is low and protein substrates are abundant [8].

Obesity, a global health crisis, is increasingly recognized as a risk modifier for both periodontitis and halitosis. Adipose tissue functions as an endocrine organ, secreting proinflammatory cytokines such as TNF-α, IL-6, and leptin that compromise host immune responses, enhance oxidative stress, and disrupt oral microbial homeostasis [14,28,29]. Studies have confirmed elevated levels of VSC and increased prevalence of *P. gingivalis* in obese individuals with periodontal disease, suggesting a synergistic pathophysiological link [17,30].

Recent findings by Alzahrani et al. (2024) revealed a significant reduction in VSC-producing bacteria following bariatric surgery, thereby reinforcing the role of obesity in modulating oral microbiota and halitosis [31]. In a similar study, authors observed alterations in periodontal parameters and microbial diversity in response to systemic cytokine-modulating therapy in obese patients with inflammatory conditions [32]. These insights underscore the importance of integrated management approaches targeting both local and systemic factors.

The essential oil mouthwash used in this study—comprising lemon (*Citrus limon*), melissa (*Melissa officinalis*), sodium bicarbonate, and grapefruit seed extract—achieved significant reductions in PI (31.5%) and BOP (34.49%), as well as a 38.09% reduction in BANA positivity. These outcomes are consistent with those of previous clinical trials, which demonstrated the antimicrobial and anti-inflammatory efficacy of essential oils in oral health applications [19,33,34].

The mechanisms through which essential oils exert their effects are diverse. These oils have been shown to disrupt bacterial membranes, inhibit quorum sensing, reduce biofilm formation, and modulate local cytokine activity [35]. The presence of citral, limonene, and geraniol contributes to their broad-spectrum antimicrobial activity. It is noteworthy that these agents also maintain biocompatibility and patient tolerability, as evidenced by the absence of mucosal irritation or taste disturbance in the present study.

These findings have been confirmed by recent meta-analyses. Dobler et al. (2020) conducted a review of the clinical outcomes of essential oil mouthrinses, confirming reductions in halitosis, plaque, and gingival indices, with fewer adverse effects than chlorhexidine (CHX) [36]. As demonstrated by Alsaffar and Alzoman (2021), the efficacy of citrus-based antioxidant mouthwashes in managing halitosis and inflammation has been substantiated [37].

In the present study, the BANA test effectively captured microbial shifts associated with VSC-producing species. The substantial correlation between the BANA test’s enhancement and the reduction in organoleptic scores validates its application as a pragmatic, cost-effective diagnostic instrument in the management of halitosis [6,38,39,40].

Chlorhexidine (CHX) is widely regarded as the gold standard for chemical plaque control. However, it is associated with several potential drawbacks, including staining, taste alteration, and mucosal irritation, particularly with prolonged use [40,41]. In contrast, essential oil formulations offer a superior safety profile with comparable clinical efficacy in mild-to-moderate inflammation, as evidenced by the findings of Van Strydonck et al. (2012) and Berchier et al. (2010) [22,23].

A significant area of research is the use of probiotics for halitosis and periodontitis management. Probiotic strains, including Streptococcus salivarius K12 and Lactobacillus reuteri, have been shown to possess the capacity to impede the proliferation of VSC-producing pathogens, bolster mucosal immune systems, and re-establish equilibrium among microbial populations [42,43]. Some studies reported improved halitosis and psychosocial outcomes using probiotic lozenges [44,45,46]. Even though the present study did not encompass probiotics, the potential exists for subsequent research to investigate the combination of probiotics and essential oils in a symbiotic approach.

Halitosis, or bad breath, has been shown to have psychosocial consequences, particularly in individuals already vulnerable to stigma due to obesity. Self-perceived halitosis has been demonstrated to exert deleterious effects on interpersonal relationships, increase social anxiety, and reduce treatment adherence [47,48].

In this study, the test group reported a 36.84% improvement in subjective halitosis scores. This finding is noteworthy in that it demonstrates not only a clinical success but also perceived psychosocial benefit. These results suggest that interventions aimed at improving breath quality may enhance patient motivation, adherence to oral hygiene protocols, and overall quality of life outcomes that should be prioritized in future research.

While the findings of this study are encouraging, it is imperative to acknowledge its limitations. Primarily, the limited 28-day duration of the study precludes the evaluation of the long-term efficacy and sustainability of clinical improvements. Future studies should include extended follow-up periods to evaluate the rates at which patients experience relapses and the duration of halitosis control treatment effectiveness. Secondly, it should be noted that the study did not incorporate advanced diagnostic methods, such as gas chromatography or sulfide monitors, to quantify volatile sulfur compounds. Instead, it relied on biogenic amine (BANA) tests and organoleptic scoring, which, while they are clinically valid, offer limited precision. The study’s limitations include its modest sample size and unequal group allocation ratio of 2:1, which may compromise the study’s generalizability. The unequal 2:1 allocation was strategically employed due to practical considerations but is recognized as a limitation. Future research should adopt balanced group sizes to improve comparative robustness. Examiner calibration was thorough, enhancing data reliability, but further methodological standardization is recommended.

Furthermore, the study failed to address the influence of uncontrolled variables on halitosis, including diet, salivary flow, and psychological stress. These variables have been demonstrated to exert a significant impact on halitosis. Finally, the test product, though effective, was a custom-made formulation not yet commercially standardized or evaluated for regulatory compliance.

Future research should prioritize the evaluation of the long-term effects and safety of essential oil-based mouthwashes through the implementation of extended follow-up studies with a duration of at least three to six months. The conducting of comparative trials against established agents such as chlorhexidine and emerging alternatives such as probiotics or symbiotic formulations may facilitate the determination of optimal adjunctive therapies. The integration of sophisticated microbial analysis methodologies, including 16S rRNA sequencing and quantitative PCR, would facilitate a more profound comprehension of the microbial shifts associated with halitosis reduction. Furthermore, the conducting of extensive multicentre trials that incorporate stratification by obesity grade, comorbid conditions, and behavioral factors (e.g., smoking, diet, oral hygiene compliance) is imperative to enhance external validity and inform personalized treatment strategies.

## 5. Conclusions

This preliminary clinical evaluation indicates that an essential oil-based mouthwash, composed of lemon, melissa, and grapefruit seed extract, has the potential to serve as an effective adjunctive treatment for reducing halitosis, plaque accumulation, gingival bleeding, and microbial burden in obese patients diagnosed with periodontitis. These findings emphasize the interplay between oral dysbiosis, systemic inflammation, and halitosis in this high-risk population. While the results are encouraging, the study’s limitations should be considered. The 28-day duration of the study, the relatively small sample size, and the reliance on semi-objective halitosis measures (e.g., BANA and organoleptic scores) are notable weaknesses. The utilization of gas chromatography and sulfide monitors was precluded due to constraints related to feasibility. The experimental mouthwash formulation was an experimental and non-commercial product.

Consequently, subsequent research endeavors should encompass larger and more heterogeneous cohorts, employ sophisticated microbiological and gas-sensing methodologies, and evaluate the long-term safety and efficacy of essential oil-based rinses in comparison to recognized agents such as chlorhexidine.

The present study, within the scope of its methodology, supports the use of essential oil mouthwashes as a potentially safe, patient-friendly approach for improving oral health and quality of life in individuals with coexisting obesity and periodontal disease.

## Figures and Tables

**Table 1:** Demographic results.

Parameter	Test Group (n = 30)	Control Group (n = 15)	*p*-Value
Mean Age (years)	43.2 ± 9.4	42.7 ± 8.8	0.81
Gender (M/F)	16/14	8/7	0.92
BMI (kg/m^2^)	32.1 ± 2.4	31.7 ± 2.3	0.63
Smokers (%)	26.7%	26.6%	0.98
Baseline PI	1.39 ± 0.15	1.33 ± 0.13	0.17
Baseline BOP (%)	58.2 ± 6.1	56.4 ± 5.7	0.31

## Data Availability

The original contributions presented in the study are included in the article, further inquiries can be directed to the corresponding author.
